# Une maladie peut cacher une autre: un mal de Pott révélant un schwannome

**DOI:** 10.11604/pamj.2014.19.91.5285

**Published:** 2014-09-26

**Authors:** Hajer Ben Brahim, Wafa Chebbi

**Affiliations:** 1 Service de Maladies Infectieuses, CHU Fattouma Bourguiba, 5000 Monastir, Tunisie; 2 Service de Médecine Interne, CHU Taher Sfar Mahdia, 5100 Mahdia, Tunisie

**Keywords:** Mal de Pott, schwannome, tumeur nerveuse, Pott disease, schwannoma, nerve tumor

## Image en medicine

Le schwannome est une tumeur nerveuse bénigne rare. Il peut être à l'origine d'une ostéolyse mécanique des pédicules des vertèbres sus et sous jacente, lors de la croissance tumorale, pour permettre l'extension extra-rachidienne. Nous rapportons une association exceptionnelle de schwannome et de mal de pott au même niveau. Il s'agissait d'une patiente âgée de 67 ans, ayant des antécédents de diabète type 2, hospitalisée pour rachialgie lombaire, d'horaire inflammatoire évoluant depuis 3 mois, associée à un amaigrissement non chiffré, une asthénie et des sueurs nocturnes. L'examen physique trouvait une patiente subfébrile à 37,8°C, une raideur rachidienne avec une douleur provoquée à la pression des épineuses de D11-D12 et à la mobilisation du rachis. L'examen neurologique était normal. L'imagerie par résonance magnétique du rachis et de la moelle, pratiquée en urgence, montrait une spondylodiscite infectieuse D11-D12 avec une épidurite antérieure et une formation tissulaire centrée sur l'angle costo-vertébral droit de D10-D11, de signal hétérogène, en hyposignal T1. Cette masse est bien limitée de 43X47x48 mm, encapsulée, se rehaussant en périphérie après injection de produit de contraste et présentant une extension au niveau du foramen intervertébral, aspect compatible. avec un schwannome. Le bilan étiologique de la spondylodiscite avait conclu à une origine tuberculeuse. Le diagnostic de schwannome était confirmé par la biopsie percutanée sous scanner. Un traitement médical par antituberculeux était entamé et la patiente est adressée en neurochirurgie. L'association au même endroit d'un mal de pott et d'une tumeur nerveuse est exceptionnelle et pose le diagnostic différentiel avec une collection paravértébrale compliquant la spondylodiscite. La greffe du Bacille de Koch à cet endroit pourrait être expliquée par les anomalies organiques provoquées lors de l'extension tumorale.

**Figure 1:**
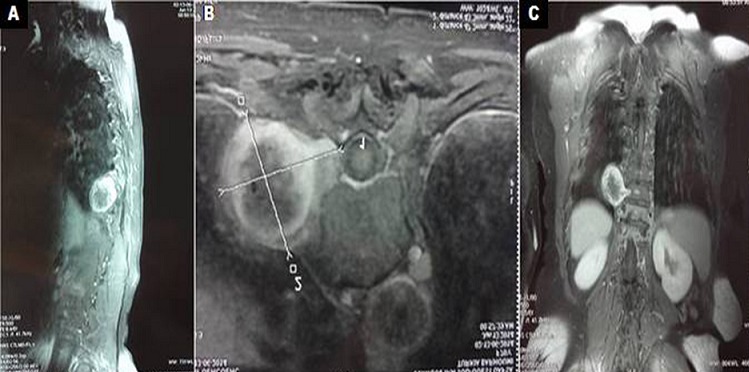
IRM séquence T1. (A): coupe sagittale; (B): coupe axiale; (C): coupe coronale. Spondylodiscite infectieuse D11-D12 avec une épidurite antérieure et une formation tissulaire centrée sur l'angle costo-vertébral droit de D10-D11, de signal hétérogène, en hyposignal T1, bien limitée de 43X47x48 mm correspondant à un schwannome

